# Accessibility of Enzymatically Delignified *Bambusa bambos* for Efficient Hydrolysis at Minimum Cellulase Loading: An Optimization Study

**DOI:** 10.4061/2011/805795

**Published:** 2011-08-29

**Authors:** Arindam Kuila, Mainak Mukhopadhyay, D. K. Tuli, Rintu Banerjee

**Affiliations:** ^1^Microbial Biotechnology and Downstream Processing Laboratory, Agricultural and Food Engineering Department, Indian Institute of Technology, Kharagpur 721 302, India; ^2^R&D Centre, Indian Oil Corporation Ltd., Faridabad 121 007, India

## Abstract

In the present investigation, *Bambusa bambos* was used for optimization of enzymatic pretreatment and saccharification. Maximum enzymatic delignification achieved was 84%, after 8 h of incubation time. Highest reducing sugar yield from enzyme-pretreated *Bambusa bambos* was 818.01 mg/g dry substrate after 8 h of incubation time at a low cellulase loading (endoglucanase, **β**-glucosidase, exoglucanase, and xylanase were 1.63 IU/mL, 1.28 IU/mL, 0.08 IU/mL, and 47.93 IU/mL, respectively). Enzyme-treated substrate of *Bambusa bambos* was characterized by analytical techniques such as Fourier transformed infrared spectroscopy (FTIR), X-ray diffraction (XRD), and scanning electron microscopy (SEM). The FTIR spectrum showed that the absorption peaks of several functional groups were decreased after enzymatic pretreatment. XRD analysis indicated that cellulose crystallinity of enzyme-treated samples was increased due to the removal of amorphous lignin and hemicelluloses. SEM image showed that surface structure of *Bambusa bambos* was distorted after enzymatic pretreatment.

## 1. Introduction

Lignocellulosic biomass, for its large quantities and relatively low cost, is regarded as the potential renewable energy resource for cost-effective bioethanol production. Lignocellulosic bioethanol production involves three major steps, including pretreatment, enzymatic hydrolysis, and fermentation. Among all these steps, efficient hydrolysis of cellulose component of lignocellulosic biomass is the key step for cost-effective bioethanol production. Cellulase is the key enzyme for hydrolysis of cellulose. *Trichoderma reesei *Rut C30 is one of the well-known cellulase (endoglucanase, *β*-glucosidase, exoglucanase, and xylanase) producing fungi. Since cellulase is still a relatively costly biocatalyst for commercially viable bioethanol production, this requires development of efficient hydrolysis of cellulose at low cellulase loading.

Lignin is key barrier which restricts the access of cellulase to cellulose. Laccase, a copper containing oxidase enzyme, can remove lignin effectively and increase the accessibility of cellulase to cellulose at mild operating conditions and minimal byproduct formation. 

*Bambusa bambos* is a rapid growing lignocellulose available in abundance on the global scenario. The bamboos are woody tree-like grasses and have a long history as an exceptionally versatile and widely used resource. India is one of the leading countries in the world in bamboo production. (4.5 million tons) [1]. *Bambusa bambos* is one of the most important species of Bamboo, which grows in India. It has high cellulose (40–50%) and moderate lignin (15–20%) content [2]. If appropriate pretreatment and saccharification strategies are performed, *Bambusa bambos *can be used as potential feedstock for cost-effective bioethanol production. Structural characteristics and chemical compounds distribution of *Bambusa bambos* before and after pretreatment could be studied using FTIR, XRD, and SEM. This could reveal structural and chemical changes that occur when particular treatments are applied to produce an optimized and high-quality product development. In recent years some researchers made progress on the utilization of Bamboo, including pretreatments and saccharification [3–5], but there is no literature on the changes in physicochemical properties for enzyme-pretreated *Bambusa bambos* and saccharification at low cellulase loading.

 In the present study, we investigated the possibility of enzymatic hydrolysis of laccase-pretreated *Bambusa bambos* at low cellulase loading. The objective of the study was to (1) optimize the laccase-mediated pretreatment of *Bambusa bambos* and evaluate the enzymatic pretreatment effect for efficient saccharification and (2) optimize the enzymatic hydrolysis of pretreated *Bambusa bambos* at low cellulase loading.

## 2. Materials and Methods

### 2.1. Lignocellulosic Substrate

*Bambusa bambos* (cellulose 47.49%, hemicellulose 17.49%, lignin 23.56%, moisture 10.23%, and ash 2.30%) was collected from local forest of IIT Kharagpur, India. It was air dried overnight at 60°C. Then it was milled to approximately 0.2 mm particle size and subsequently used for further studies.

### 2.2. Enzymes

Laccase and cellulase were produced from *Pleurotus* sp. and *Trichoderma reesei* Rut C30, respectively [6, 7]. Laccase and cellulase were centrifuged at 5,000 rpm for 5 minutes. The clear filtrate of laccase was used as crude laccase whose activity was determined spectrophotometrically [6]. One international unit/mL (IU/mL) of laccase activity was defined as the amount of enzyme capable of oxidizing 1 *μ*mol of ABTS per minute under the assay conditions. Clear supernatant of cellulase (pH 5) was used as crude cellulase whose activity was determined by following assay protocols [8, 9]. One international unit/mL (IU/mL) of cellulase activity was defined as the amount of enzyme required to produce 1 *μ*mol of glucose per minute under the assay conditions. The activity of endoglucanase, *β*-glucosidase, exoglucanase, and xylanase were 16.34 IU/mL, 12.80 IU/mL, 0.78 IU/mL, and 479.33 IU/mL, respectively.

### 2.3. Enzymatic Pretreatment and Saccharification of *Bambusa bambos*

Enzymatic pretreatment of *Bambusa bambos *was performed in Erlenmeyer flask, containing 10 g of substrate, 0.1 mol/L of phosphate buffer (pH 6.5 to 7.5) and required volume of laccase. Samples were withdrawn periodically, and the solid residue was used for lignin estimation. After delignification the solid residue was washed with distilled water. Then it was air dried overnight at 60°C and subsequently used for further studies.

For enzymatic hydrolysis, pretreated sample of *Bambusa bambos* was taken in Erlenmeyer flask containing 18 mL of 0.1 mol/L of phosphate buffer (pH 4 to 8) and 2 mL of cellulase enzyme. Sample aliquots were taken periodically and centrifuged at 2,000 rpm for 5 minutes. The supernatant was analyzed for reducing sugar by following dinitrosalicylic acid method [10]. The extent of hydrolysis was calculated as follows:

(1) $$\;\;\begin{array}{cc} & \text{Saccharification}\;\left(\text{\%}\right)\\ & \;=100\times [\text{Reducing}\;\text{sugar}\;\text{concentration}\;\text{obtained}/\;\\ & \;\;\;\;\;\;\;\;\;\;\text{Potential}\;\text{sugar}\;\text{concentration}\;\\ & \;\;\;\;\;\;\;\;\;\text{in}\;\text{the}\;\text{pretreated}\;\text{substrate}]. \end{array}$$

### 2.4. Response Surface Methodology

Response Surface Methodology- (RSM-) based three level Central Composite Design (CCD) was employed for optimization of enzymatic pretreatment and saccharification of *Bambusa bambos*. For pretreatment of* Bambusa bambos*, five parameters were selected in the range pH (6.5–7.5), temperature (35–45°C), liquid : solid ratio (2–6 mL/g), incubation time (4–8 h), and enzyme concentration (400–600 IU/gds). For saccharification of pretreated *Bambusa bambos*, four parameters were selected in the range of pH (4–8), substrate concentration (16–20 mL/g), temperature (40–60°C), and incubation time (4–8 h). All the pretreatment and saccharification experiments were carried out in triplicates. In coded terms the lowest, central, and the highest level of the variables were −1, 0, and +1, respectively. Tables 1 and 2 show the pretreatment and saccharification result, respectively. 

### 2.5. Analytical Methods

#### 2.5.1. FTIR, XRD, and SEM Study of *Bambusa bambos*

Fourier transformed infrared spectroscopy (FTIR) was performed in both the original and pretreated samples using the KBr pellet technique. Sample spectra were obtained over the range of 400 cm^−1^ and 4000 cm^−1^ with a spectral resolution of 0.5 cm^−1^. 

The crystallinity of original and pretreated sample were determined by XRD1710 equipment using CoK_*α*_ radiation (*α* = 1.79 Å) at 40 kV and 20 mA. All samples were scanned from 2*θ* = 15 to 75° with scanning speed of 3°/min. Crystallinity (%) was defined as [(*I*_002_ − *I*_am_)/*I*_002_] × 100, where *I*_002_ represent maximum crystalline intensity peak at 2*θ* between 22° and 23° for cellulose *I*, and *I*_am_ representss minimum crystalline intensity peak at 2*θ* between 18° and 19° for cellulose *I* [11].

To analyze surface characteristics of original and pretreated samples, Scanning electron microscopic (SEM) image was taken for both original and pretreated sample of *Bambusa bambos*. For SEM, dried sample was coated with gold and observed under JEOL JSM 5800 (Jeol Ltd., Tokyo, Japan) SEM.

## 3. Result and Discussion

### 3.1. Optimization of Enzymatic Pretreatment and Saccharification of *Bambusa bambos*

Using the designed experimental data (Table 1), the second-order polynomial model for the percent delignification in terms of coded factors is shown as follows:

(2) $$\begin{array}{cc} Y_{1} & =-2314.71+639.76A_{1}+44.65A_{2}\;+191.75A_{3}\\ & \;-5.27A_{4}+58.34A_{5}-43.48A_{1}^{2}-0.10A_{2}^{2}\\ & \;-{2.00A}_{3}^{2}-{4.29A}_{5}^{2}-7.17A_{1}A_{2}-17.10A_{1}A_{3}\\ & \;+0.59A_{1}A_{4}+4.10A_{1}A_{5}-2.10A_{2}A_{3}+0.05A_{2}A_{4}\\ & \;-0.09A_{2}A_{5}+0.10A_{3}A_{4}-3.28A_{3}A_{5}-0.02A_{4}A_{5}, \end{array}$$

where *Y*_1_ is the percent delignification and *A*_1_, *A*_2_, *A*_3_, *A*_4_, and *A*_5_ represent pH, temperature, liquid : solid ratio, enzyme concentration, and incubation time, respectively.

Whereas, using the experimental data (Table 2) the second-order polynomial model for the reducing sugar production (mg/gram dry substrate) of pretreated *Bambusa bambos*, in terms of coded factors, is shown as follows which are also given in coded terms:

(3) $$\begin{array}{cc} Y_{2}\; & =-4626.87+260.67B_{1}+17.35B_{2}+433.93B_{3}\\ & \;+82.19B_{4}-{16.20B}_{1}^{2}-{\;0.16B}_{2}^{2}-{11.29B}_{3}^{2}\\ & \;-{0.73B}_{4}^{2}-0.73B_{1}B_{2}-0.37B_{1}B_{3}-3.21B_{1}B_{4}\\ & \;+0.06B_{2}B_{3}+0.15B_{2}B_{4}-3.43B_{3}B_{4}, \end{array}$$

where the variables *B*_1_, *B*_2_, *B*_3_, and *B*_4_ represent pH, temperature, substrate concentration, and incubation time, respectively, and *Y*_2_ represents reducing sugar yield (mg/gram dry substrate) from pretreated substrate.

Based on the experimental response, for enzymatic pretreatment, runs 12 and 31 had the minimum and maximum delignification, respectively, whereas, for saccharification of enzyme pretreated substrate, runs 3 and 31 had the minimum and maximum reducing sugar production, respectively. The ANOVA results of second-order response surface models for enzymatic pretreatment and saccharification of *Bambusa bambos* have been given in Table 3. From ANOVA analysis of regression model, for enzymatic pretreatment of *Bambusa bambos* at 20 degree of freedom, *F*-value was 22.07 and *P* value was <0.001. For saccharification of enzyme-pretreated substrate *F* and *P* values were 64.72 and <0.001, respectively, at 20 degree of freedom. For both enzymatic pretreatment and saccharification of *Bambusa bambos F*-values were several times higher than the *P* values. So, *F* and *P* values indicated that the quadratic regression models for enzymatic pretreatment and saccharification of *Bambusa bambos* were significant, and insignificant *F* and *P* values of lack of fit for models indicated that experimental data obtained were in good agreement with the model [12]. The goodness of fit of regression models was checked by the determination coefficient (*R* ^2^). The coefficients of determination (*R* ^2^) were calculated to be 0.9757 and 0.9816 for enzymatic pretreatment and saccharification, respectively. For a good statistical model, *R* ^2^ value should be close to 1.0.

**Figure 1:**
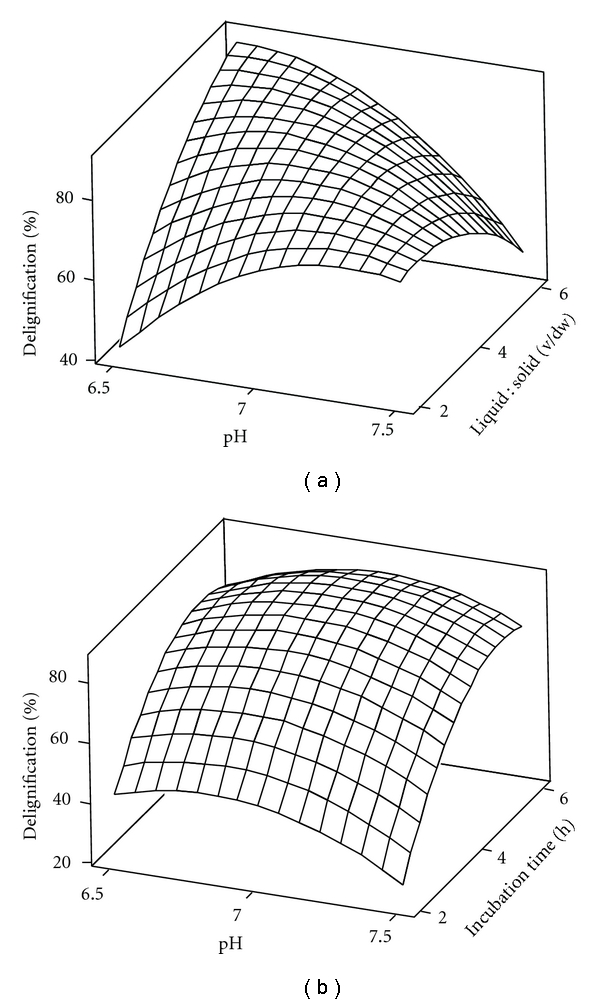
RSM plot showing (a) the effect of pH and liquid : solid ratio and (b) the effect of pH and incubation time on enzymatic pretreatment of *Bambusa bambos*.

The 3D response surface plots are generally the graphical representation of the regression equation. Figures 1(a) and 1(b) represent the 3D response surface plot for the optimum conditions of enzymatic delignification of *Bambusa bambos*. Each figure represents effect of two variables on percent delignification. From the analysis of the response surface plots, the optimum conditions for enzymatic delignification were pH 6.87, temperature 35.26°C, liquid : solid ratio 6 : 1, incubation time 8 h, and enzyme concentration 400 IU/mL. Under the optimum conditions, the maximum predicted delignification was 83%, which was close to the experimental response (84%). Reducing sugar yield could be significantly increased by removing about 80% of lignin from lignocellulosic biomass [13].

**Figure 2:**
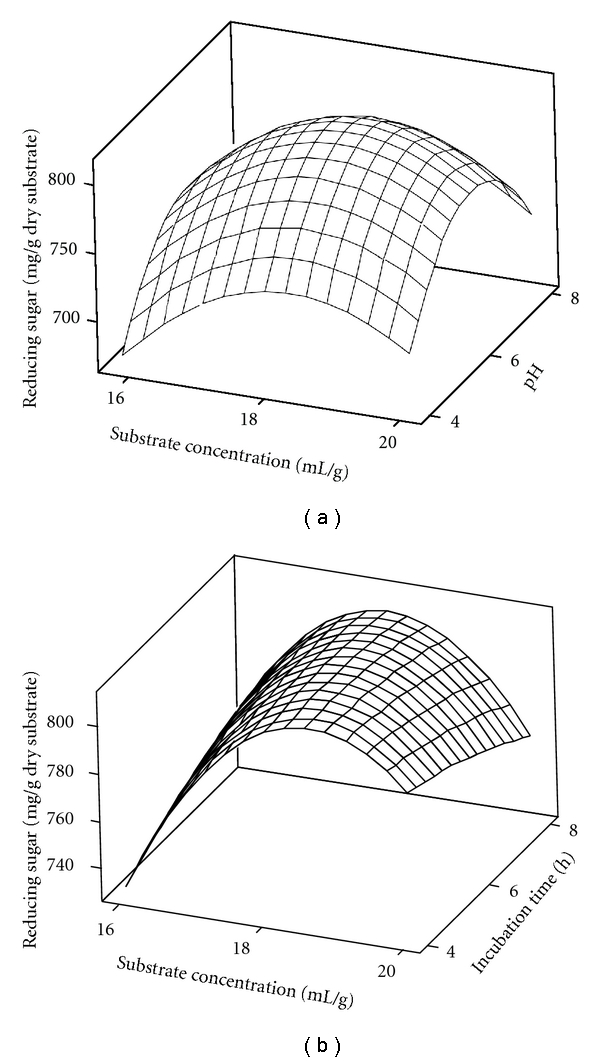
RSM plot showing (a) the effect of substrate concentration and pH and (b) substrate concentration and incubation time on saccharification of enzyme-pretreated *Bambusa bambos*.

Figures 2(a) and 2(b) present the 3D response surface plots for the optimization conditions of saccharification of enzyme-pretreated substrate.  Figure 2(a) shows the effect of substrate concentration (mL/g) and pH, and Figure 2(b) shows the effect of substrate concentration (mL/g) and incubation time (h) on saccharification. From response surface plot analysis, under optimum conditions (pH 6, temperature 50°C, substrate concentration 18 mL/g, and incubation time of 8 h) the experimental maximum reducing sugar yield was 818.01 mg/g dry substrate which was close to predicted response. At low cellulase loading (endoglucanase, *β*-glucosidase, exoglucanase, and xylanase were 1.63 IU/mL, 1.28 IU/mL, 0.08 IU/mL, and 47.93 IU/mL, respectively) the enhancement of saccharification (reducing sugar yield) reported here is much greater than that cited in other literatures [12, 14]. It was also reported that physical and chemical pretreatment generates several toxic compounds which decrease the rate of saccharification as well as reducing sugar yield [15].

Similar reducing sugar yield (818.01 mg/g dry substrate) was reported by other authors at high cellulase loading [16, 17]. Cost-effective biofuel production needs low cellulase loading and higher reducing sugar yield in short incubation time [18]. The saccharification efficiency (71.28%) obtained was in agreement with earlier reports [19, 20].

There are so many reports on using commercial cellulase for saccharification of different lignocellulosic biomass and also further addition of several additives was required [14, 21]. In the present study, pretreatment was performed with crude laccase, and saccharification was performed with crude cellulase enzyme without addition of any additives. 

### 3.2. Physical Properties of Pretreated *Bambusa bambos*

**Figure 3:**
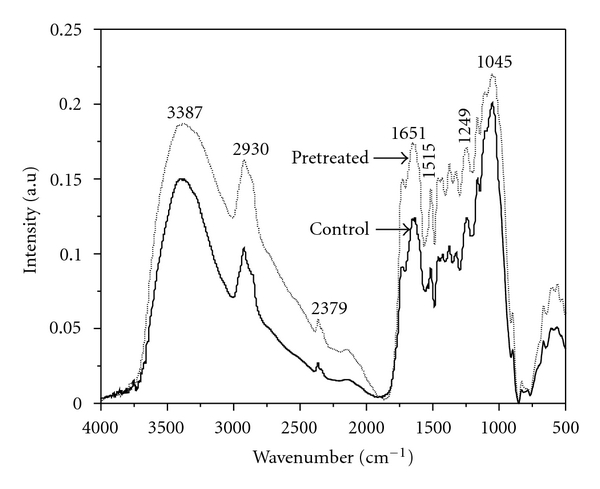
FTIR spectra of control and enzyme-pretreated *Bambusa bambos*.

FTIR analysis was taken to qualitatively observe the changes of functional groups and further evaluate component modification. Significant changes in FTIR spectra could be seen after enzymatic pretreatment of *Bambusa bambos* (Figure 3). It shows that intensity at 3387 cm^−1^ (OH vibration), 2930 cm^−1^ (C–H methyl and methylene groups), 2379 cm^−1^ (C=O bonds in ketene groups), 1651 cm^−1^ (C=C stretching), 1515 cm^−1^ (C=C aromatic), 1249 cm^−1^ (O–H phenolic) and 1045 cm^−1^ (lignin) [22–25] were decreased after enzyme pretreatment. The reduced intensity indicates cleavage of lignin side chains [26]. These results highlight the effectiveness of enzymatic pretreatment for efficient saccharification of *Bambusa bambos*. 

**Figure 4:**
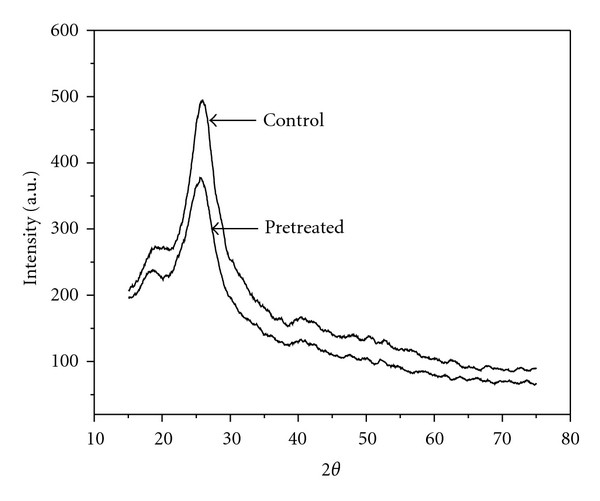
XRD diagram of control and enzyme-pretreated *Bambusa bambos*.

Lignocellulosic biomass is mainly composed of cellulose, hemicellulose, and lignin. Among several effecting factors, crystallinity is believed to significantly affect enzymatic saccharification of cellulose [27]. However, because of the impossibility of completely separating cellulose from other components of the fibers, the direct measurements of cellulose crystallinity in biomass were hindered [13]. The X-ray diffraction patterns of control and pretreated samples are presented in Figure 4. It was found that cellulose crystallinity of control and pretreated samples were 28.44% and 33%, respectively. These results indicate effective pretreatment on *Bambusa bambos* which removes amorphous hemicellulose and lignin and expose all crystalline cellulose available and increase the rate of enzymatic saccharification [28].

**Figure 5:**
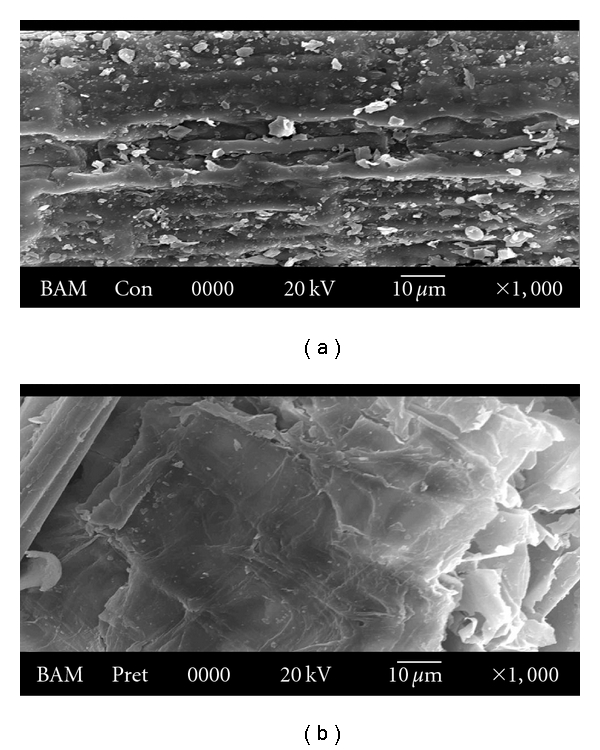
SEM view of (a) control and (b) enzyme-pretreated sample of *Bambusa bambos*.

SEM was used to determine changes in surface structure of *Bambusa bambos *after enzymatic pretreatment. SEM image of *Bambusa bambos* before and after enzymatic pretreatment is shown in Figure 5. It shows that surface structure in the control *Bambusa bambos* was rigid and highly ordered (Figure 5(a)), while the structure was distorted after pretreatment (Figure 5(b)). It was due to degradation of lignin after enzymatic pretreatment, which increased the surface area of cellulose, making it more accessible to cellulase [29].

## 4. Conclusion

Enzymatic pretreatment and saccharification of *Bambusa bambos* were carried out using laccase and cellulase enzyme, respectively. The conditions for enzymatic pretreatment and saccharification of *Bambusa bambos* were optimized by using RSM-based CCD. Using laccase optimum delignification (84%) was achieved after 8 h of incubation time. Maximum reducing sugar yield (818.01 mg/gram dry substrate) from enzyme-pretreated substrate was attained after 8 h of incubation time at low cellulase loading. FTIR, XRD, and SEM study further revealed the effectiveness of enzymatic pretreatment for efficient saccharification of *Bambusa bambos*.

## Figures and Tables

**Table 1:** Experimental design (conditions and responses) for enzymatic pretreatment of *Bambusa bambos *in terms of coded factor.

Run order	*A*_1_	*A*_2_	*A*_3_	*A*_4_	*A*_5_	Delignification (%)	
						Experimental	Predicted
(1)	−1 (6.5)	−1 (35)	−1 (2)	−1 (400)	+1 (8)	75.33	73.807
(2)	+1 (7.5)	−1 (35)	−1 (2)	−1 (400)	−1 (6)	62.41	63.282
(3)	−1 (6.5)	+1 (45)	−1 (2)	−1 (400)	−1 (4)	43.36	41.834
(4)	+1 (7.5)	+1 (45)	−1 (2)	−1 (400)	+1 (8)	45.13	44.381
(5)	–1 (6.5)	–1 (35)	+1 (6)	−1 (400)	−1 (4)	82.64	82.938
(6)	+1 (7.5)	−1 (35)	+1 (6)	−1 (400)	+1 (8)	38.7	39.775
(7)	–1 (6.5)	+1 (45)	+1 (6)	−1 (400)	+1 (8)	41.73	40.843
(8)	+1 (7.5)	+1 (45)	+1 (6)	+1 (600)	−1 (4)	51.78	52.852
(9)	+1 (7.5)	−1 (35)	−1 (2)	+1 (600)	+1 (8)	63.29	64.066
(10)	−1 (6.5)	+1 (45)	−1 (2)	+1 (600)	+1 (8)	55.53	54.345
(11)	+1 (7.5)	+1 (45)	−1 (2)	+1 (600)	−1 (4)	50.69	50.392
(12)	−1 (6.5)	−1 (35)	+1 (6)	+1 (600)	+1 (8)	35.6	36.239
(13)	+1 (7.5)	−1 (35)	+1 (6)	+1 (600)	−1 (4)	83.1	84.626
(14)	−1 (6.5)	+1 (45)	+1 (6)	+1 (600)	−1 (4)	80.19	79.318
(15)	+1 (7.5)	+1 (45)	+1 (6)	+1 (600)	+1 (8)	62.34	62.68
(16)	−1 (6.5)	0 (40)	0 (4)	0 (500)	0 (6)	68.2	73.255
(17)	+1 (7.5)	0 (40)	0 (4)	0 (500)	0 (6)	71.09	66.477
(18)	0 (7)	−1 (35)	0 (4)	0 (500)	0 (6)	82.37	78.707
(19)	0 (7)	+1 (45)	0 (4)	0 (500)	0 (6)	73.41	77.515
(20)	0 (7)	0 (40)	−1 (2)	0 (500)	0 (6)	63.11	66.744
(21)	0 (7)	0 (40)	+1 (6)	0 (500)	0 (6)	81.9	78.709
(22)	0 (7)	0 (40)	0 (4)	−1 (400)	0 (6)	63.4	65.84
(23)	0 (7)	0 (40)	0 (4)	+1 (600)	0 (6)	80.2	78.203
(24)	0 (7)	0 (40)	0 (4)	0 (500)	−1 (4)	45.12	44.92
(25)	0 (7)	0 (40)	0 (4)	0 (500)	+1 (8)	80.72	82.234
(26)	0 (7)	0 (40)	0 (4)	0 (500)	0 (6)	82.6	80.736
(27)	0 (7)	0 (40)	0 (4)	0 (500)	0 (6)	74.7	80.736
(28)	0 (7)	0 (40)	0 (4)	0 (500)	0 (6)	80.05	80.736
(29)	0 (7)	0 (40)	0 (4)	0 (500)	0 (6)	81.9	80.736
(30)	0 (7)	0 (40)	0 (4)	0 (500)	0 (6)	83.44	80.736
(31)	0 (7)	0 (40)	0 (4)	0 (500)	0 (6)	84.1	80.736
(32)	0 (7)	0 (40)	0 (4)	0 (500)	0 (6)	81.0	80.736

**Table 2:** Experimental design (conditions and responses) for enzymatic saccharification of pretreated *Bambusa bambos *in terms of coded factor.

Run order	*B*_1_	*B*_2_	*B*_3_	*B*_4_	Reducing sugar (mg/g of substrate)	
					Experimental	Predicted
(1)	−1 (4)	−1 (40)	−1 (16)	+1 (8)	671.21	671.15
(2)	+1 (8)	−1 (40)	−1 (16)	−1 (6)	691.45	698.26
(3)	−1 (4)	+1 (60)	−1 (16)	−1 (4)	621.67	632.60
(4)	+1 (8)	+1 (60)	–1 (16)	+1 (8)	639.10	656.21
(5)	−1 (4)	−1 (40)	+1 (20)	−1 (4)	695.47	683.00
(6)	+1 (8)	−1 (40)	+1 (20)	+1 (8)	697.92	691.63
(7)	−1 (4)	+1 (60)	+1 (20)	+1 (8)	700.13	705.95
(8)	+1 (8)	+1 (60)	+1 (20)	−1 (4)	701.25	705.95
(9)	+1 (8)	–1 (40)	−1 (16)	+1 (8)	698.32	693.32
(10)	−1 (4)	+1 (60)	−1 (16)	+1 (8)	702.23	692.28
(11)	+1 (8)	+1 (60)	−1 (16)	−1 (4)	657.13	647.90
(12)	−1 (4)	−1 (40)	+1 (20)	+1 (8)	667.27	675.42
(13)	+1 (8)	−1 (40)	+1 (20)	−1 (4)	741.71	750.58
(14)	−1 (4)	+1 (60)	+1 (20)	−1 (4)	696.09	696.60
(15)	+1 (8)	+1 (60)	+1 (20)	+1 (8)	660.43	659.35
(16)	−1 (4)	0 (50)	0 (18)	0 (6)	734.01	735.68
(17)	+1 (8)	0 (50)	0 (18)	0 (6)	767.35	751.44
(18)	0 (6)	−1 (40)	0 (18)	0 (6)	798.66	798.65
(19)	0 (6)	+1 (60)	0 (18)	0 (6)	801.12	786.90
(20)	0 (6)	0 (50)	−1 (16)	0 (6)	758.24	747.63
(21)	0 (6)	0 (50)	+1 (20)	0 (6)	782.41	778.79
(22)	0 (6)	0 (50)	0 (18)	0 (6)	801.23	808.36
(23)	0(6)	0(50)	0 (18)	0(6)	797.39	808.36
(24)	0 (6)	0 (50)	0 (18)	−1 (4)	808.56	805.25
(25)	0 (6)	0 (50)	0 (18)	+1 (8)	809.72	805.61
(26)	0 (6)	0 (50)	0 (18)	0 (6)	801.43	808.36
(27)	0 (6)	0 (50)	0 (18)	0 (6)	795.12	808.36
(28)	0 (6)	0 (50)	0 (18)	0 (6)	811.06	808.36
(29)	0 (6)	0 (50)	0 (18)	0 (6)	810.06	808.36
(30)	0 (6)	0 (50)	0 (18)	0 (6)	802.62	808.36
(31)	0 (6)	0 (50)	0 (18)	0 (6)	817.31	808.36
(32)	0 (6)	0 (50)	0 (18)	0 (6)	803.08	808.36

**Table 3:** ANOVA analysis of RSM model for enzymatic pretreatment of *Bambusa bamboos *and saccharification of pretreated *Bambusa bambos. *

Source	DF^a^	Seq SS^b^	Adj SS^b^	Adj MS^c^	*F*	*P*
Pretreatment of *Bambusa bamboos *						

Regression	20	7547.10	7547.10	502.796	22.07	<0.001
Linear	5	1115.70	1395.38	375.443	16.32	<0.001
Square	5	2592.68	3959.72	473.038	46.32	<0.001
Interaction	10	3838.72	3838.72	383.872	22.45	<0.001
Residual error	11	188.05	188.05	17.096		
Lack-of-fit	5	128.62	128.62	25.724	2.60	0.138
Pure error	6	59.44	57.44	9.906		

Total	32	7735.16				
*R* ^2^ = 97.57%, *R* ^2^ = 93.15%						

Saccharification of pretreated *Bambusa bamboos *						

Regression	14	116738	116737.6	8338.4	64.72	<0.001
Linear	4	6116	22570.9	5642.7	43.80	<0.001
Square	4	103427	98888.2	24722.0	191.90	<0.001
Interaction	6	7195	7194.6	1199.1	9.31	< 0.001
Residual error	17	2190	2190.1	128.8		
Lack-of-fit	9	1788	1788.0	198.7	3.95	0.033
Pure error	8	402	402.1	50.3		

Total	32	118928				
*R* ^2^ = 98.16%, *R* ^2^ = 96.64%						

^a^Degrees of freedom.

^b^Sum of squares.

^c^Mean squares.
